# Supplementary material for: Social Relationships, Age and the Use of Preventive Health Services: Findings from the German Ageing Survey
Source: Int J Environ Res Public Health. 2019 Nov 4;16(21):4272. doi: 10.3390/ijerph16214272 (PMC6862648; doi:10.3390/ijerph16214272)
Supplement: Supplementary file 1 [file ijerph-16-04272-s001.pdf]

Logistic regression models for flu vaccination (Model 1.1) and cancer screening (Model 2.1) with interaction terms between informational support and age (German Ageing Survey, 2014).

	Seasonal flu vaccination (Model 1.1)		Cancer screening (Model 2.1)	
Predictors	*Odds Ratios*	*95% CI*	*Odds Ratios*	*95% CI*
**(Intercept)**	0.01	0.00-0.04	0.00	0.00-0.00
**Gender (Ref. male): female**	1.03	0.93-1.15	2.37	2.14-2.63
**Age in years**	1.06	1.04-1.08	1.38	1.17-1.63
**Informational support (Ref. no): yes**	1.28	0.38-4.50	5.77	0.03-1598.26
**ISCED-1: low**	0.89	0.75-1.06	1.00	1.00-1.00
**ISCED-3: high**	1.03	0.93-1.15	0.87	0.73-1.04
**Number of physical diseases**	1.08	1.04-1.11	1.13	1.02-1.26
**Very good**	0.50	0.40-0.62	1.05	1.01-1.08
**Good**	0.73	0.65-0.82	0.78	0.64-0.94
**Bad**	1.07	0.89-1.29	1.06	0.94-1.19
**Very bad**	1.69	1.20-2.40	0.77	0.64-0.93
**Partner (Ref. no): yes**	1.20	1.07-1.34	0.77	0.55-1.08
**Social network size (number of important persons with regular contact)**	0.99	0.97-1.01	1.57	1.41-1.75
**Age*informational support**	1.00	0.98-1.02	1.02	1.00-1.04
**Age in years (cubic term)**			0.98	0.82-1.15
**Age in years (cubic term)*informational support**			1.00	1.00-1.00
**Observations**	7,588		7,515	

Logistic regression models for flu vaccination (Model 1.2) and cancer screening (Model 2.2) with interaction terms between having a partner and age (German Ageing Survey, 2014).

	Seasonal flu vaccination (Model 1.2)		Cancer screening (Model 2.2)	
Predictors	*Odds Ratios*	*95% CI*	*Odds Ratios*	*95% CI*
**(Intercept)**	0.01	0.01-0.02	0.00	0.00-0.00
**Gender (Ref. male): female**	1.03	0.93-1.14	2.44	2.19-2.70
**Age in years**	1.06	1.05-1.07	1.31	1.23-1.39
**Informational support (Ref. no): yes**	1.42	0.78-2.58	0.52	0.04-7.02
**ISCED-1: low**	0.89	0.74-1.06	1.00	1.00-1.00
**ISCED-3: high**	1.03	0.93-1.15	0.87	0.73-1.05
**Very good**	1.08	1.04-1.11	1.14	1.02-1.26
**Good**	0.50	0.40-0.62	1.05	1.02-1.08
**Bad**	0.73	0.65-0.82	0.78	0.64-0.95
**Very bad**	1.07	0.89-1.29	1.07	0.95-1.20
**Partner (Ref. no): yes**	1.70	1.20-2.40	0.77	0.64-0.93
**Social network size (number of important persons with regular contact)**	1.38	1.13-1.70	0.76	0.54-1.08
**Age*partner**	0.99	0.97-1.01	1.41	1.16-1.71

**Age in years (cubic term)**			1.02	1.00-1.04
**Age in years (cubic term)*partner**			1.02	0.94-1.11
**Observations**	7,588		7,515	

Logistic regression models for flu vaccination (Model 1.3) and cancer screening (Model 2.3) with interaction terms between social network size and age (German Ageing Survey, 2014).

	Seasonal flu vaccination (Model 1.3)		Cancer screening (Model 2.3)	
Predictors	*Odds Ratios*	*95% CI*	*Odds Ratios*	*95% CI*
**(Intercept)**	0.01	0.01-0.02	0.00	0.00-0.04
**Gender (Ref. male): female**	1.03	0.93-1.15	2.38	2.15-2.64
**Age in years**	1.06	1.05-1.07	1.20	1.10-1.31
**Informational support (Ref. no): yes**	1.00	0.90-1.11	0.51	0.31-0.82
**ISCED-1: low**	0.89	0.74-1.06	1.00	1.00-1.00
**ISCED-3: high**	1.03	0.93-1.15	0.87	0.73-1.04
**Very good**	1.08	1.04-1.11	1.14	1.02-1.27
**Good**	0.50	0.40-0.62	1.05	1.01-1.08
**Bad**	0.73	0.65-0.82	0.79	0.65-0.95
**Very bad**	1.07	0.89-1.29	1.07	0.95-1.20
**Partner (Ref. no): yes**	1.69	1.20-2.40	0.78	0.65-0.94
**Social network size (number of important persons with regular contact)**	1.38	1.13-1.70	0.77	0.55-1.09
**Age*network size**	1.20	1.07-1.34	1.40	1.15-1.69
**Age in years (cubic term)**			1.58	1.41-1.76
**Age in years (cubic term)*network size**			1.02	1.01-1.04
**Observations**	7,588		7,515	
